# Dichoptic Game Training in Strabismic Amblyopia Improves the Visual Evoked Response

**DOI:** 10.7759/cureus.45395

**Published:** 2023-09-17

**Authors:** Emmanouil Blavakis, Jenny Spaho, Marina Chatzea, Angleliki Gleni, Sotiris Plainis

**Affiliations:** 1 Ophthalmology, Jules Gonin Eye Hospital, University of Lausanne, Lausanne, CHE; 2 Laboratory of Optics and Vision, School of Medicine, University of Crete, Heraklion, GRC; 3 Optometry, Optical House, Heraklion, GRC

**Keywords:** vision training, dichoptic training, vep, strabismus, amblyopia

## Abstract

Dichoptic video gaming offers an alternative approach in amblyopia treatment by allowing different information to be presented in the two eyes, resulting to reduced suppression and/or enhanced fusion. The aim of this case report series is to evaluate the outcome of supervised dichoptic training, with the use of video games in a virtual reality (VR) system, on far and near visual acuity (VA), stereoacuity, and the visual evoked response of an adult and two children with strabismic amblyopia. Results suggest that despite the absence of improvement in VA following supervised dichoptic training, a remarkable increase in stereoacuity was evident with a concurrent decrease in phorias. Moreover, an improvement in the P100 latency of the pattern visual evoked potentials (VEPs) in the amblyopic eye was observed in all participants. Finally, at least two sessions per week were completed for each patient under continuous supervision, implying sufficient compliance and treatment efficiency with dichoptic video gaming. Supervised dichoptic training, consisting of at least 20 hours of video gaming using a VR system, improves stereoacuity and the latency of the visual evoked response in the amblyopic eye. This probably occurs by overcoming its suppression, indicating that the speed of visual processing, as evaluated by pattern VEPs, may precede improvements in VA.

## Introduction

Amblyopia is a neurodevelopmental visual dysfunction, leading to a unilateral or bilateral reduction in best-corrected visual acuity (BCVA), not attributable to an observable structural or organic cause [1]. It is caused by the presence of at least one amblyopia risk factor early in life, including anisometropia, strabismus, and stimulus deprivation (induced by congenital cataract for example), estimated to affect 1% to 5% of the population worldwide [2].

Although amblyopia is clinically diagnosed by reduced BCVA in at least one eye, most amblyopia patients also exhibit significant impairment in binocular vision and stereopsis [3,4], and contrast sensitivity [3-5], showing also subtle deficits in reading performance [6,7] and depth perception [3], essential for real-world visuomotor tasks [4]. Thus, the early detection and management of amblyopia is considered of utmost importance, and the earlier the treatment, the better the chance of resolution [8]. Traditionally, amblyopia caused by strabismus and anisometropia is managed before the first seven to eight years of age, because during this period, due to the plasticity of the visual system, most visual deficits are more likely to be managed effectively [1,9]. While amblyopia is more difficult to treat with increasing age, treatment is also encouraged in older children and teenagers [2].

The standard approach in the management of amblyopia is the correction of any refractive error present followed by occlusion, bangerter filters, or atropine penalization of the non-amblyopic eye [1,2]. New treatments have been implemented recently in the clinical practice, based on purely binocular viewing [10]. These involve dichoptic stimulation and manipulation of contrast in video games with the aim to reduce suppression and/or improve fusion upon training [11-13]. In such a setting, to overcome suppression, the amblyopic eye receives a more intense stimulus than the fellow eye, by reducing the contrast and/or the luminance of the image presented in the fellow eye [12].

This unmasked prospective case report evaluates the effect of dichoptic training on three patients using standard visual function measures, such as visual acuity (VA) and stereoacuity. In addition, supra-threshold performance was investigated by measuring response amplitude and latency in pattern-reversal visual evoked potentials (pVEPs).

## Case presentation

Supervised dichoptic training was performed using Vivid Vision software (San Francisco, USA), which includes custom-made action video games using a virtual reality (VR) headset (Oculus Rift VR headset, Oculus VR, California, USA). Each training session lasted one hour and was repeated two to four times per week. During the session, each patient played five games, aiming to alleviate suppression (the first four) or targeted to the training of fusion/stereoscopic vision (the last one). The games consisted of different key elements and surroundings with increasing difficulty during game play, while they included settings for misalignment correction. To reduce suppression of the amblyopic eye and facilitate fusion, a perceptual balance task was performed, where the images perceived by the two eyes were presented dichoptically, and the contrast of the image seen by the fellow eye was modulated. Initially, an image with full contrast was presented to the amblyopic eye and a black screen to the fellow eye. The contrast of the image for the fellow eye was gradually increased in 10% steps, by the clinician, until the patient perceived two equally contrast-balanced images. This procedure was repeated three times; the average value was used to play the game. All three patients managed to complete at least two sessions per week during their supervised training period, considered of sufficient compliance and treatment efficiency with dichoptic video gaming. Written informed consent was obtained for identifiable health information used in this case report.

A standard orthoptic examination was performed before and after completion of dichoptic training, with the best-spectacle correction and room lights on (illuminance at cornea was 75 lux), which included (a) monocular and binocular distant and near VA using the European-wide modified logMAR charts (Precision Vision, USA) at 4 m and 40 cm, respectively; (b) stereoacuity, using the Randot® Stereo Test (Stereo Optical, US) at 40 cm; and (c) the alignment with the alternating prism cover test at 4 m and 40 cm distances.

Monocular pVEPs were elicited using reversing 30 and 10 arcmin checks (nominal dominant spatial frequency 1 and 3 cpd), with the latter known to be most sensitive to contrast changes [14,15], at a rate of four reversals (2 Hz) per second with square-wave modulation. The stimulus, displayed on a Sony GDM F-520 CRT monitor, subtended a circular field of 15 degrees (at a 1.0 m testing distance) with 100% contrast and a mean luminance of 30 cd/m^2^. Fixation was achieved using a centrally placed cross. VEPs were recorded using silver-silver chloride electrodes. An active electrode was positioned 10% of the distance between the inion and the nasion over the vertex and referenced to an electrode placed at Fz with a ground electrode placed on the forehead. The active and reference electrodes were applied to the head with electrode paste after the area had been thoroughly cleaned. Trigger synchronization was achieved using a CED 1401 “micro” (Cambridge Electronic Design, UK). The waveforms were amplified (gain=10K) using the CED 1902 (Cambridge Electronic Design, UK). The amplifier bandwidth was set at 0.5-30 Hz (together with a 50 Hz notch filter), and signals were sampled at a rate of 1024 Hz with an analysis time of 0.970 s. Data acquisition and averaging were controlled using the Signal 4.1 software (CED, UK). Each VEP trace was the average of 64 epochs of one second duration each, as suggested by the International Society of Clinical Electrophysiology of Vision (ISCEV). The P100 peak amplitude and latency (time) were derived from the average waveform. Amplitude was scored as the voltage difference between the lowest negative peak (N75) prior to the P100 peak and latency as the time difference between the P100 peak and stimulus onset.

Case 1

**Figure 1:**
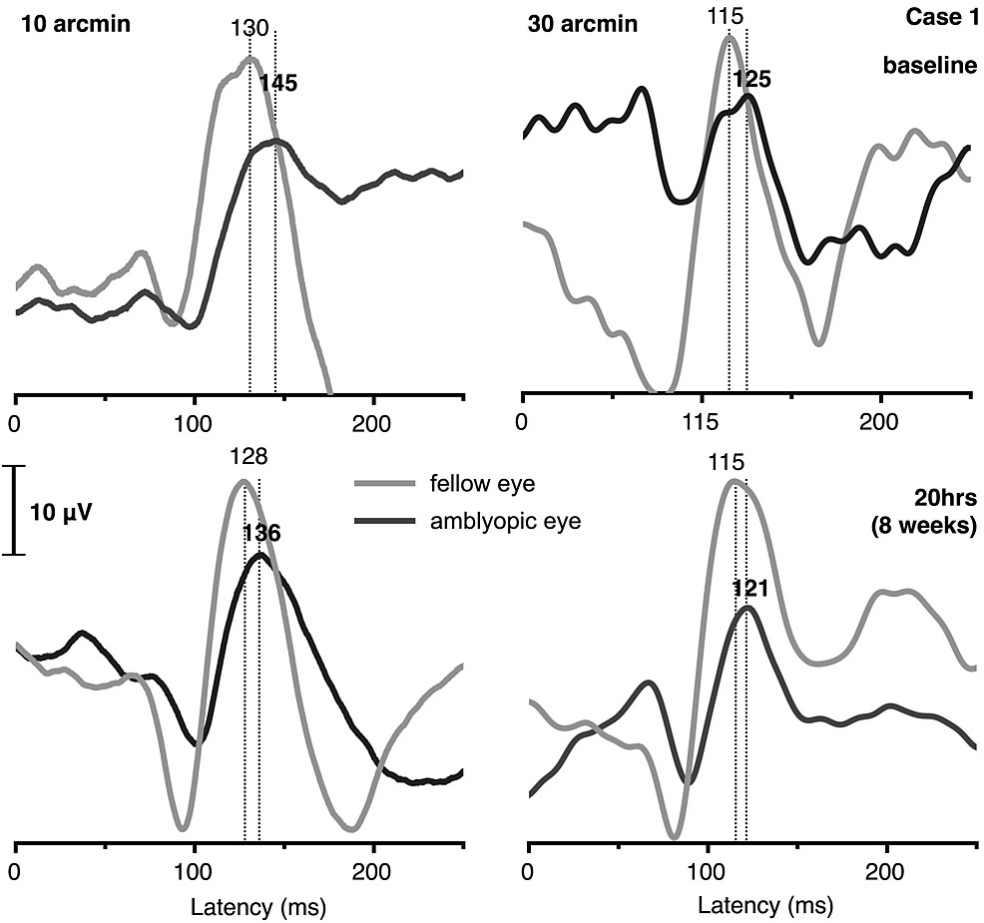
Grand-averaged (64 epochs) monocular pVEP waveforms for case 1 at 15 arcmin (left) and 40 arcmin (right) size, elicited using 2 Hz-reversing check pattern of 100% contrast for the amblyopic eye (black line) and the fellow eye (gray line), before treatment (baseline), and after 20 hours (eight weeks) of dichoptic training. P100 latency is indicated in ms.

A nine-year-old girl presented with hypermetropia and anisometropia (RE +3.50D, LE +5.50D), an esophoria of 16Δ for far when uncorrected (4Δ with spectacle correction), and a BCVA of 0.28 logMAR in her left amblyopic eye (-0.02 logMAR in the fellow eye) (see Table 1). She completed 20 one-hour sessions of supervised dichoptic training over a period of eight weeks, without any significant changes in her far and near monocular and binocular BCVA. Her stereoacuity with spectacles improved from 100 to 50 arcsec. In addition, esophoria improved to 1Δ with spectacle correction and to 8Δ without correction. Her monocular pVEP waveforms are plotted in Figure 1. The latency of the pVEP P100 peak was found to be improved in the amblyopic eye after the training, with the effect being more pronounced at 10 arcmin (from 145 ms to 136 ms) than the 30 arcmin check size (from 125 to 121 ms). The P100 amplitude was found improved following the training at 30 arcmin (from 10.7 to 15.4 μV), while no difference was observed at 10 arcmin (from 16.1 to 15.5 μV).

**Table 1:** Best-corrected visual acuity (BCVA) at distance and at near and stereo acuity at baseline and following dichoptic training for all three cases.

BCVA (logMAR)	Amblyopic eye	Fellow eye	Binocularly	Stereo acuity (arc sec)
Case 1				
Baseline				
Distance	0.28	-0.02	0.00	
Near	0.34	0.06	0.00	100
8 weeks (20 sessions)				
Distance	0.28	0.00	0.00	
Near	0.32	0.06	-0.02	50
Case 2				
Baseline				
Distance	0.36	0.02	0.00	
Near	0.44	0.02	0.00	400
8 weeks (20 sessions)				
Distance	0.42	0.00	0.00	
Near	0.42	0.04	0.00	40
Case 3				
Baseline				
Distance	0.32	-0.14	-0.14	
Near	0.34	-0.10	-0.16	400
5 weeks (20 sessions)				
Distance	0.24	-0.24	-0.24	
Near	0.30	-0.10	-0.12	60
12 weeks (50 sessions)				
Distance	0.32	-0.24	-0.24	
Near	0.32	-0.10	-0.12	40

Case 2

**Figure 2:**
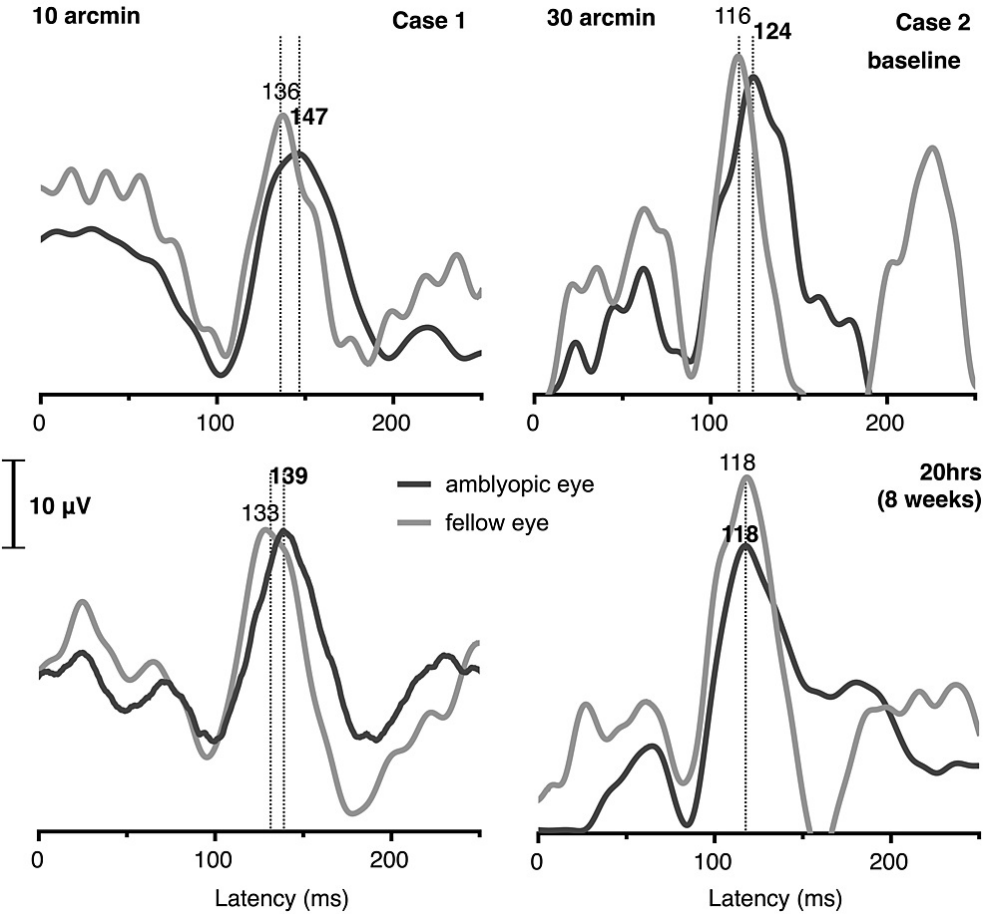
Grand-averaged (64 epochs) monocular pVEP waveforms for case 2 at 15 arcmin (left) and 40 arcmin (right) size, elicited using 2 Hz-reversing check pattern of 100% contrast for the amblyopic eye (black line) and the fellow eye (gray line), before treatment (baseline), and after 20 hours (eight weeks) of dichoptic training. P100 latency is indicated in ms.

A nine-year-old boy presented with low anisometropia (RE +1.75D/-0.50Dx5, LE +0.50D), microstrabismus (4Δ for far), and a BCVA of 0.32 logMAR in his right amblyopic eye (0.02 logMAR in the fellow eye) (see Table 1). He followed 20 one-hour sessions of supervised dichoptic training over a period of eight weeks. No significant differences were observed in his far and near binocular and monocular BCVA and in the alignment at 4 m. However, stereoacuity improved significantly from 400 to 50 arcsec. The latency of the P100 peak in the amblyopic eye was found improved after training, from 147 ms to 139 ms at 10 arcmin and from 124 to 118 ms at 30 arcmin (see Figure 2). No significant differences after training were found in the P100 amplitude.

Case 3

A 24-year-old male with a history of strabismic amblyopia, two surgeries for correction of strabismus (at the the age of six and 18 years, respectively), and absence of any refractive error, presented with a 20Δ esophoria at far, a stereoacuity of 400 arc seconds, and a BCVA of 0.32 logMAR in his amblyopic eye (0.14 logMAR in the fellow eye). The patient completed 50 one-hour sessions of dichoptic training over a period of three months. Although an improvement of 0.08 logMAR in distance and near VA was observed at five weeks in the amblyopic eye, it did not persist following 12 weeks of training (see Table 1). His esophoria at far improved to 16Δ and his stereoacuity to 40 arcsec.

**Figure 3:**
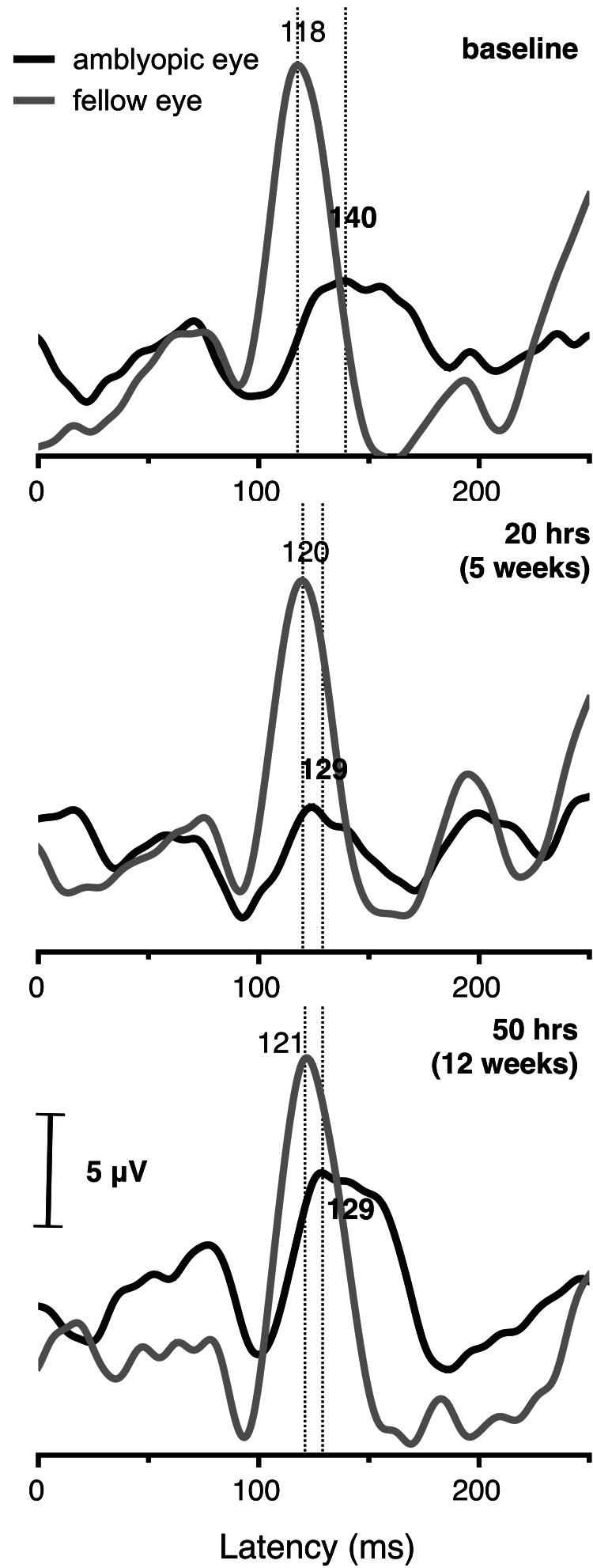
Grand-averaged (64 epochs) monocular pVEP waveforms for case 3, elicited using 2 Hz-reversing 10 arcmin checks of 100% contrast for the amblyopic eye (black line) and the fellow eye (gray line) before treatment (baseline), after 20 hours (five weeks), and 50 hours (12 weeks) of dichoptic training. P100 latency is indicated in ms.

The latency of the P100 peak in the amblyopic eye was improved from 140 ms to 129 ms, after five weeks of training with no further improvement at 12 weeks (129 ms). The P100 amplitude showed no difference after five weeks of training (from 3.7 to 3.9 μV) but improved to 6.1 μV after 12 weeks (see Figure 3).

## Discussion

The results of the study suggest that supervised dichoptic training using a VR system improves stereoacuity and the visual evoked response in the amblyopic eye, in the absence of any improvement in VA. A possible explanation for the lack of a gain in VA in mild amblyopia, in agreement with previous studies [11,13], could be the limited image resolution of the VR headset, which cannot display stimuli better than 0.5 logMAR, failing to provide a high-resolution experience for patients with better acuity. It is a viable hypothesis that gains in acuity are more likely when the headset resolution is better than patients’ BCVA.

It is well established that the standard treatment of amblyopia, with occlusion or penalization of the fellow eye, focuses on improving VA [1,2,13]. On the contrary, dichoptic gaming is targeted to overcome suppression of the amblyopic eye, ﻿by presenting a weak but visible (contrast-balanced) stimulus to the fellow dominant eye to enhance fusion/stereoscopic vision [10]. It is hypothesized that dichoptic binocular training using a VR headset could allow the visual system, due to its plasticity, to continue learning from the binocular experience outside of the VR, using the high-resolution retinal images provided by the real world. Although this was not observed in the present study, there are reports of a 0.1-0.2 logMAR benefit in acuity just from overcoming suppression [12].

Dichoptic training also produced a notable gain in stereoacuity, reaching functional levels. Impaired stereoscopic depth perception is the most common deficit associated with amblyopia under ordinary (binocular) viewing conditions, usually with a substantial impact on visuomotor tasks [7], having a positive impact in patients’ quality of life. Note that stereopsis is much more impacted, and recovery may require more active treatment in strabismic than in anisometropic amblyopia [16].

The improvement in the P100 latency and amplitude of the pVEPs in the amblyopic eye following dichoptic training is not unexpected; pVEPs have been used as a suprathreshold measure of early pre-cortical visual processing, i.e., contrast attenuation and transfer, revealing binocular facilitation effects [14,15]. It has also been suggested that in the presence of inhibitory conditions, such as amblyopia, binocular interaction is hampered [17,18]; thus, it is not surprising that dichoptic training effects transfer to the fundamental components of visual perception, such as contrast sensitivity, in the amblyopic eye [19]. Facilitatory interaction between the signals from the two eyes is further supported by electrophysiological studies in animals, demonstrating a direct relationship between the reduced sensitivity of foveal cortical cells receiving input from the amblyopic eye and contrast sensitivity [20].

As amblyopia may recur in up to 25% of patients during the first year of treatment discontinuation [8], long-term follow-up is required to determine the overall effectiveness of dichoptic training. In addition, further research should be conducted using standardized and reproducible methods of visual acuity and stereoacuity assessment. Besides evaluating the efficacy of binocular treatments, future randomized controlled trials could also focus on the use of dichoptic training as an adjuvant treatment to monocular therapies, such as cover treatment.

## Conclusions

Although VA examination forms the standard test in the diagnosis of amblyopia, the main efficacy outcomes following standard intervention therapies, dichoptic stimulation, and manipulation of contrast in video games are not targeted specifically to acuity. This case series shows that dichoptic gaming, of at least 20 one-hour sessions of video gaming using a VR system, improves stereoacuity and the latency of the visual evoked response in the amblyopic eye. This probably occurs by overcoming its suppression, indicating that the speed of visual processing, as evaluated by pVEPs, may precede improvements in VA.
